# Electrospinning of botanicals for skin wound healing

**DOI:** 10.3389/fbioe.2022.1006129

**Published:** 2022-09-19

**Authors:** Shijie Guo, Pengyu Wang, Ping Song, Ning Li

**Affiliations:** ^1^ Department of Biomedical Engineering and Technology, Institute of Basic Theory for Chinese Medicine, China Academy of Chinese Medical Sciences, Beijing, China; ^2^ Department of Dermatology, Guang’anmen Hospital, China Academy of Chinese Medical Sciences, Beijing, China

**Keywords:** botanical, electrospinning, wound healing, wound dressing, nanofiber

## Abstract

Being the first barrier between the human body and external environments, our skin is highly vulnerable to injuries. As one of the conventional therapies, botanicals prepared in different topical formulations have been applied as medical care for centuries. With the current increase of clinical requirements, applications of botanicals are heading towards nanotechnologies, typically fused with electrospinning that forms nanofibrous membranes suitable for skin wound healing. In this review, we first introduced the main process of wound healing, and then presented botanicals integrated into electrospun matrices as either loaded drugs, or carriers, or membrane coatings. In addition, by addressing functional features of individual botanicals in the healing of injured skin, we further discussed the bioactivity of botanical electrospun membranes in relevant to the medical issues solved in the process of wound healing. As achieved by pioneer studies, due to infrequent adverse effects and the diversity in resources of natural plants, the development of electrospun products based on botanicals is gaining greater attention. However, investigations in this field have mainly focused on different methodologies used in the preparation of nanofibrous membranes containing botanicals, their translation into clinical practices remains unaddressed. Accordingly, we propose that potential clinical applications of botanical electrospun membranes require not only the further expansion and understanding of botanicals, but also an establishment of standard criteria for the evaluation of wound healing and evolutions of technologies to support the large-scale manufacturing industry.

## 1 Introduction

The skin is the first physical barrier of human body that serves a variety of pivotal functions, from preventing external pathogens to conveying somatosensory signals and regulating temperature and hydration. Due to a direct contact with external environments, our skin is extremely labile to injuries, which often lead to bacterial infections and even life-threatening complications. To facilitate the natural healing process, wound dressings that provide favorable environmental conditions for the healing of skin injuries are generally required. Conventionally, cream, gauze, and cotton wool are the major management for wound care; however, their poor air permeability, short residence time, uncontrollable drug dosages, and the resultant infections have put forward the evolution of wound dressings much more than just a topical cover. Currently, wound-care technologies are tremendously expanding to nanotechnology engineering, the application of which has shown excellent outcomes (Kalashnikova et al., 2015). Among different nanomaterials, nanofibers prepared by electrospinning have been extensively implemented as optimal dressing materials owing to their superior physical and mechanical properties. In particular, the high porosity, small diameters and the large surface area have endowed electrospun nanofibers with core competencies, such as excellent breathability and effective control of drug release (Yang and Leong, 2010; Ye and KuangYouMorsiMo, 2019; Hernandez and Woodrow, 2022). Moreover, the relatively low cost and the ease of manufacture have made electrospun nanofibers suitable for a large-scale production.

Botanicals have been developed and utilized as medical care for centuries similar to that of modern pharmaceuticals. Compared to pharmaceutical drugs, botanical ingredients integrated into dermatological preparations are witnessing sharp popularity because of their infrequent adverse effects and diversities in resources and bioactive ingredients (Fuhrmann et al., 2010). Though increasingly attractive, the ancient producing process of topical formulations in phytomedicine has hindered the development and generalization of phytotherapies. In this scenario, a variety of studies have attempted to formulate botanical wound dressings by incorporating electrospinning technologies. In that way, benefits in air permeability, moisture maintenance, exudes absorption, controlled drug release, and the subsequent reduction of infections have been achieved (Sundaramurthi et al., 2014).

**FIGURE 1:**
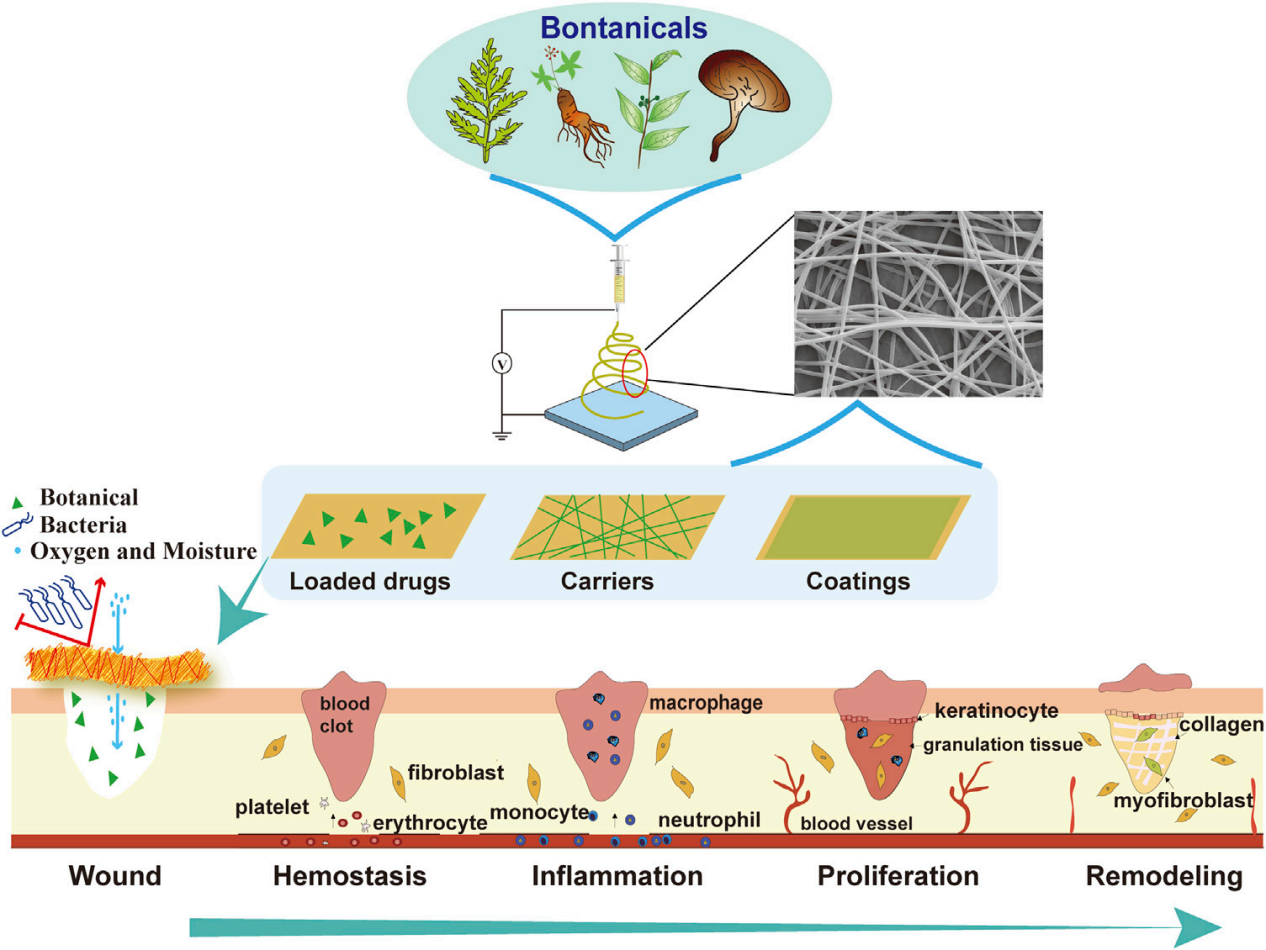
Electrospinning of botanicals for skin wound healing. A variety of botanicals can be electrospun as wound dressings in the form of loaded drugs, or carriers, or coatings, to provide a suitable microenvironment for different stages of wound healing, including hemostasis, inflammation, proliferation and remodeling.

Hence, this review aims to present the progress in the fundamental research of electrospun nanofibers with the application of botanicals, focusing on the advanced methodologies employed and medical issues solved during the process of wound healings (Figure 1). Moreover, to put forward the clinical applications of botanical-containing electrospun nanofibers, we propose strategic suggestions to optimize the roadmap of botanical electrospun products.

## 2 Wound healing

### 2.1 The process of wound healing

Wound healing is a dynamic and highly regulated process of cellular, humoral and molecular mechanisms, comprising a series of pathophysiological alterations of four phases, which include hemostasis, inflammation, proliferation, and remodeling (Wilkinson and Hardman, 2020). In general, immediately after injury, blood vessels around the wound undergo reactive contractions, with aggregations of platelets due to collagen exposure, leading to the formation blood clots and extracellular matrix at the injury site. The local necrosis and release of vasoactive substances often cause acute inflammatory responses, as manifested as congestion, plasma exudation, and leukocyte excretion. During this stage, injured cells and pathogens are generally removed from the wound area by immune cells, through mechanisms induced by phagocytosis and reactive oxygen species (ROS). Meanwhile, two opposite activations of macrophages known as the pro-inflammatory M1 and the tissue-remodeling M2 are initiated, with the former gradually converts into the latter to drive wound healings from inflammatory responses to the proliferation phase (Kang et al., 2018; Kim et al., 2019). During the proliferation step, fibroblasts migrate, proliferate, and secrete large amounts of collagen and extracellular matrix, resulting in the formation of granulation tissues (Driskell et al., 2013). When the growing of granulation tissues persists, the skin remodeling is consequently triggered, which is characterized by the development of scar tissues that have disturbed alignment of collagen fibers and excessive dermal fibrosis. Therefore, the complex process of wound healing requires a suitable microenvironment that sequentially functions to repair damaged tissues and facilitates the overall restoration of skin function and texture (Guo and Dipietro, 2010; Broussard and Powers, 2013).

### 2.2 Current wound dressings

With the recognition of the complexity of wound healings, wound dressings have evolved from traditional passive dressings in the available forms of gauze, bandages, and cotton wool, to interactive dressings like films, foams, and hydrogels, then to modern bioactive dressings that are produced by biomaterials (Aderibigbe and Buyana, 2018; Farahani and Shafiee, 2021). Passive dressings, which are non-occlusive, are used to cover wounds and absorb exudates. Since absorptions of wound exudates often result in secondary injuries due to the adhesion of dressings to the damaged area, they practically fulfill few protections (Fraccalvieri et al., 2011). The interactive dressings, which are designed based on interactions between dressings and wounds, are semi-occlusive or occlusive (Mostafalu et al., 2018). Practically, interactive dressings are preferred as a physical barrier against microorganisms in addition to facilitations of healing by enhancing exudates absorption and gas exchange. As for bioactive dressings, their production involves combinations of polymer materials and bioactive substances with high-tech approaches. Compared to the first two forms, bioactive dressings, such as electrospun membranes, have shown their advantages in terms of retaining moisturization and delivering medicinal materials which are conducive to accelerate the healing process (Sood et al., 2014).

## 3 Electrospinning of botanicals for skin wound healing

### 3.1 Electrospun nanofibers

Electrospun nanofibers are organized filaments that are fabricated under a strong electric field force. The innate properties of electrospun matrix like the high surface-to-volume ratio and porosity in structure have enabled nanofibrous mats suitable for wound healing (Rieger et al., 2013). Besides, by modulating the arrangement of nanofibers, the controlled release of loaded drugs and the superior mechanical properties which potentially assist to adapt to limb activities can be realized. Regarding materials used for electrospinning, various polymers can be selected according to specific requirements of impaired skin. For example, the poly (lactic-co-glycolic acid), polycaprolactone and silk fibroin as scaffolds provide favorable mechanical properties, and materials like hyaluronic acid, chitosan and polyethylene oxide are polymers with antibacterial effects (Wang et al., 2020a; Ionescu et al., 2021; Luo et al., 2021; Sun et al., 2021; Wu et al., 2022; Zamboni et al., 2023). Besides, the alginate, poly (vinyl alcohol) and gelatin as absorbent polymers are selected to remove wound exudates and keep the wound moist (Yang et al., 2021; Yang et al., 2022). In addition, electrospun nanofibers formed by polymers have favorable biocompatibility and biodegradability, which practically aid in reducing biological heterogeneities (Sheikholeslam et al., 2018; Soares et al., 2018). Overall, the unique advantages of electrospun nanofibers have appeared to be ideal solutions to solve existing problems of topical therapeutics.

### 3.2 Incorporative strategies of botanicals into electrospun nanofibers

#### 3.2.1 As loaded drugs

The slow onset of therapeutic effects and a higher effective dosage needed due to low bio-availabilities of botanical components in conventional topical formulations constitute the main obstacles preventing the successfully clinical translation of botanicals (Reuter et al., 2010). Though the introduction of electrospinning technology has shown its value in optimizing the unsatisfactory features of botanicals as topical treatments, the poor water solubility and low chemical stability of botanicals have substantially curtailed their loading potentials into nanofibers. To effectively load botanicals into electrospun membranes, both needleless and needle systems have been reported, the latter of which is currently the predominant methodology and can be further categorized as blend, coaxial and multi-jet according to the number of needles installed and parameters set (Table 1).

**TABLE 1:** Botanicals as loaded-drugs used in electrospinning for wound healing.

Bioactive agent	Polymers	Electrospinning design	Hydrophilicity		Drug release (%)^a^	Tensile properties (MPa)	Diameter (nm)	Properties for wound healing	References
			Water contact angles (°)	Swelling index (%)					
anemoside B4	chitosan/polyvinyl alcohol	blend	31.1 (1 s)	N/A	70–80 (10 h)	N/A	150–250	1. Anti-inflammation	Zhang et al. (2022a)
								2. Antioxidation	
								3. Proliferation: re-epithelization/collagen matrix deposition/angiogenesis	
								4. Prominent water absorption	
								5. Biomimetic elastic mechanical properties	
								6. Sustained release of anemoside B4	
dihydroquercetin	chitosan/polyvinylpyrrolidone	Blend	10.847 (2 s)	N/A	N/A	N/A	532 ± 75	1. Antibacteria: *Staphylococcus aureus*/*Escherichia coli*	Zhang et al. (2022b)
								2. Antioxidation	
								3. Proliferation: re-epithelization/angiogenesis	
								4. Favorable morphology	
								5. Thermal stability	
								6. Hydrophilicity	
epigallocatechin-3-O-gallate	poly (L-Lactic-co-caprolactone)/gelatin	coaxial	53	N/A	about 36 (12 h) 86 (72 h)	4.5^b^	295	1. Hemostasis	Li et al. (2022)
								2. Antibacteria: *Staphylococcus aureus*/*Escherichia coli*	
								3. Antioxidation	
								4. Excellent biocompatibility	
								5. Hydrophilicity	

asiaticoside	polyvinyl alcohol/sodium alginate/silk fibroin	blend/crosslinked (glutaraldehyde)	N/A	100 (480 min)	60–80 (24 h)	20.65 ± 1.79^c^	100–140	1. Antibacteria: *Pseudomonas aeruginosa*/*Staphylococcus aureus*	Anand et al. (2022)
								2. Proliferation: re-epithelization/collagen matrix deposition	
								3. Permeability	
								4. Sustained release of asiaticoside	
								5. Wettability	
								6. Extendibility	
emodin	polyvinylpyrrolidone/cellulose acetate	Coaxial core-shell	N/A	N/A	85.55 ± 0.67 (12 h)	N/A	692 ± 93 (shell) 223 ± 31 (core)	1. Antibacteria: methicillin-resistant *Staphylococcus aureus*	Ye et al. (2020)
								2. Sustained release of emodin	
								3. No cytotoxicity	
sesamols	cellulose acetate/zein	blend	36.5 (5 s)	N/A	Ethanol: about 70 (20 min)/90 (120 min) Water: 70 (24 h)	N/A	150–250	1. Anti-inflammation	Fl, (2020)
								2. Proliferation: angiogenesis	
								3. Small diameter	
								4. Uniform distribution	
								5. Stable intermolecular structure	
								6. Low infiltration speed	
								7. High stability in water	
astragulus polysaccharide	poly (lactide-co-glycolide)	blend	N/A	N/A	N/A	N/A	570 ± 120	1. Proliferation: angiogenesis	Yang et al. (2015)
								2. High drug-loading capacity	
								3. Long lasting pharmacological effects	
baicalein	silk fibroin/polyvinylpyrrolidone	blend	N/A	N/A	silk fibroin/baicalein: about 30 (24 h) silk fibroin/polyvinylpyrrolidone/baicalein: about 65 (24 h)v	N/A	267 ± 40	1. Antibacteria: *Staphylococcus aureus*	Chan et al. (2017)
								2. Anti-inflammatory	
								3. Proliferation: angiogenesis/synthesis of collagen fibers	
								4. Sustained release of baicalein	
thymol^d^	silk fibroin/polycaprolactone/hyaluronic acid	blend/crosslinked (dried under vacuum at room temperature)/multi-layer	103.10 ± 6.57 (top layer) 38.77 ± 5.32 (bottom layer)	42 (PBS, pH = 8)	91.87 ± 0.99 (PBS, pH = 8)	7.59 ± 1.26	471.4 ± 151.6 (top layer) 295.4 ± 88.4 (bottom layer)	1. Antibacteria: *Pseudomonas aeruginosa*/*Staphylococcus aureus*	Miguel et al. (2019)
								2. Antioxidation	
								3. Proliferation: fibroblast adhesion and proliferation	
								4. Permeability	
								5. Biocompatibility	
								6. Wettability	
								7. Extendibility	
quercetin	poly (lactic acid)/graphene oxide	blend	N/A	N/A	89.96 (300 min) 87.69 (1 min/50 Hz)	1.661 ± 1.469	1,100 ± 210	1. Faster quercetin released	Croitoru et al. (2021)
								2. Electro-responsive drug delivery system	
								3. Biocompatibility	
								4. Anti-inflammation	
								5. Proliferation: fibroblast adhesion	
curcumin^e^	chitosan/gelatin/polycaprolactone/polyethylene oxide/silk fibroin	blend/three layers	95.6 (10 s/s layer)	450–500	about 40 (pH = 6.8/24 h)	N/A	556 ± 82 (second layer)	1. Suitable water absorption capacity and water vapor transmission rate	Chen et al. (2021)
								3. Hemostasis	
								4. Antibacteria: *Staphylococcus aureus*/*Escherichia coli*	
								5. Antioxidation	
								6. Anti-inflammation	
								7. Proliferation: epidermal regeneration/collagen deposition	
Chamomile extract^f^	polycaprolactone/carboxyethyl chitosan/polyvinyl alcohol	blend/crosslinked -glutaraldehyde/three layers	136.1 ± 3.1 (first layer) 41.8 ± 1.0 (third layer)	N/A	65 (5 h) 18 (336 h)	9.1 ± 1.01 (third layer)	248 ± 30 (first layer) 149 ± 33 (third layer)	1. Antibacteria: *Staphylococcus aureus*/*Escherichia coli*	Shokrollahi et al. (2020)
								2. Antioxidation	
								3. Favorable morphology	
								4. Extendibility	
								5. Sustained drug release	
*Isatis* root extract	Polyvinylpyrrolidone	blend (hand-held electrospinner)	5.35	N/A	N/A	N/A	1710 ± 820	1. Antibacteria: *Staphylococcus aureus*/*Escherichia coli*	Dong et al. (2020)
								2. Easiness in operation	
*Centella asiatica* extract	gelatin/polyvinyl alcohol	blend/crosslinked (glutaraldehyde)	44 ± 4	N/A	N/A	N/A	150–350	1. Antibacteria: *Staphylococcus aureus*/*Escherichia coli*/*Pseudomonas aeruginosa*	Yao et al. (2017)
								2. Proliferation: fibroblast proliferation/synthesis of collagen fibers	
								3. Hydrophilicity	
								4. Biodegradability	
*Gymnema sylvestre* extracts^g^ (core) minocycline hydrochloride (shell))	gelatin (core) polycaprolactone/gelatin (shell)	coaxial/core-shell	40.3 ± 5.1 (USE/minocycline hydrochloride) 38.3 ± 4.5 (CME/minocycline hydrochloride)	N/A	50 (USE/minocycline hydrochloride): 3.3 days 50 (CME/minocycline hydrochloride): 2.1 days	4.3 ± 1.1 (USE/minocycline hydrochloride) 3.5 ± 1 (CME/minocycline hydrochloride)	302 ± 44 (USE/minocycline hydrochloride) 340 ± 64 (CME/minocycline hydrochloride)	1. Antibacteria: methicillin-resistant *Staphylococcus aureus*/*Pseudomonas aeruginosa*	Ramalingam et al. (2021)
								2. Proliferation: re-epithelization/collagen matrix deposition	
								3. Favorable morphology	
								4. Wettability	
								5. Enhanced mechanical properties	
								6. Drug synergy	
								7. Sustained drug release	
*Lawsonia inermis*	l-polylactic acid/gelatin	Blend	N/A	N/A	about 60 (10 h)	N/A	520.7 ± 196	1. Antibacteria: *Staphylococcus aureus*/*Escherichia coli*	Vakilian et al. (2018)
								2. Sustained drug release	
								3. Stable drug structure	
*Agrimonia eupatoria* L. extract	polyvinyl alcohol/chitosan	blend/two layers (cotton bandage gauze &amp; polyvinyl alcohol/*Agrimonia eupatoria* L. extract/chitosan)	42.37 ± 7.52 (polyvinyl alcohol/*Agrimonia eupatoria* L. extract/chitosan)	400 (pH = 5.5) 320 (pH = 8.0)	72.18 ± 3.71 (pH = 5.5/6 h) 62.68 ± 3.87 (pH = 8.0/6 h)	26.55 ± 1.41	208.11 ± 57.92 (polyvinyl alcohol/*Agrimonia eupatoria* L. extract/chitosan)	1. Antibacteria: *Staphylococcus aureus*/*Pseudomonas aeruginosa*	Mouro et al. (2020)
								2. Biocompatibility	
								3. Wettability	

a Drug release assays were performed by default in PBS (pH = 7).

b All specimens (50 mm × 10 mm, n = 5) were tested with a crosshead speed of 10 mm min−1 until breakage.

c The ends of the samples were attached to the tensile testing machine’s gripping units, and a load of 10 kN was applied at a rate of 1 mm/min until the samples broke.

d Thymol is added to the silk fibroin and hyaluronic acid of the bottom layer.

e Curcumin was incorporated into polycaprolactone as the second layer.

f Chamomile extract was added to the second and third layers.

g *GSylvestre* leaves were extracted by ultrasonic-assisted extraction and cold immersion extraction to obtain two extracts, abbreviated as USE, and CME, respectively.

##### 3.2.1.1 Blending electrospinning

Blending electrospinning involves the mixture of more than two materials into one solution, of which only one material solution is electrospinnable. Typically, in the case when botanicals are not able to be electrospun, electrospinnable polymers are generally added as a companion. For instance, as one of the main components of clove oil, which has wound-healing effects, eugenol is unstable and poorly soluble (Banerjee et al., 2020; Nisar et al., 2021). By designing cyclodextrin inclusion complexes using electrospinning, an enhanced thermal stability and water solubility of eugenol was obtained (Celebioglu et al., 2018). In addition to advances in physical performance, electrospinning-based botanical nanofibers generally exhibit a biphasic mode of drug release. Since the initial burst release caused by drug diffusion from the fiber surface may enable a rapid production of required pharmacological responses, and the subsequent sustained drug release from the interior of nanostructures is beneficial to maintain a similar bioactivity over extended periods, the biphasic mode of drug release provides more flexible controls for botanicals with various therapeutic effects. For example, to take full advantages of antioxidant and anti-inflammatory properties of curcumin, healing potentials of curcumin-loaded silk fibroin nanofibers in combination with hydrophobic polycaprolactone and hydrophilic polyvinyl alcohol (PVA) has been investigated in diabetic wounds. *In vitro* assessment showed that approximately 20–28% curcumin release occurred within the first 1 h, followed by an increased drug release to 53–75% at 12 h. This biphasic curcumin release was proved essential to restore the normal skin structure of diabetic wounds (Agarwal et al., 2021).

##### 3.2.1.2 Coaxial electrospinning

Considering that some drugs are susceptible to denaturation or inactivation in organic solvents, core-shell nanofibers produced by coaxial electrospinning have been established (Sun et al., 2019). In the double-layer structure of core-shell nanofibers, unstable botanicals can be preserved in the core, while the shell could be utilized to carry stable components according to specific therapeutic demands. The other contribution of coaxial electrospinning is the arrangement of sequential drug release by adjusting either the thickness of shells or the chemical properties of polymers. For example, lidocaine hydrochloride has been constructed with chitosan/polyethylene oxide (PEO) as the shell for the relief of the pain, while anti-inflammatory curcumin was introduced into the core composed of polycaprolactone. *In vitro* drug release analysis showed that about 21.31% lidocaine release could be detected from the shell at 0.5 h, which was significantly higher compared to 8.89% curcumin released from the core. Then, after 72 h, when lidocaine release (57.43%) tended to be stable, the detected free curcumin raised to 68.24%. The sequential drug release from coaxial nanofibers was shown to provide both immediate analgesic effects and long-term antibacterial activities. Interestingly, the controlled drug release could be additionally achieved by adjusting the pH of solutions, because chitosan is soluble in acidic environments. Furthermore, since sodium bicarbonate (NaHCO_3_) reacts with hydrogen ions to form CO_2_, the addition of NaHCO_3_ to the curcumin/polycaprolactone core potentially promoted formations of fiber pores leading to enhanced curcumin discharge (Guo et al., 2020).

##### 3.2.1.3 Multi-jet electrospinning

In multi-jet electrospinning, different solution systems are proposed to work simultaneously to promote multi-drug delivery and physical properties of fibrous membranes. As illustrated in the treatment of chronic diabetic ulcers, to enhance mechanical and thermal stabilities of nanofibrous scaffolds loaded with antibacterial cerium oxide nanoparticles (nCeO_2_), a dual spinneret electrospinning technique involving fabrications of PVA/gelatin incorporated with nCeO_2_ and polyurethane containing cinnamon essential oil was arranged (Hussein et al., 2022). With additions of cinnamon essential oil, the strength of scaffolds was augmented from 5.16 ± 0.41 to 9.12 ± 1.52 MPa, which met the most suitable tensile strength criterion of wound healing (Cui et al., 2009). Besides, the multi-jet electrospinning has been employed to facilitate industrial large-scale productions of nanofibers, though several practical issues have been raised recently (Kumar et al., 2010; Wu et al., 2018; Vass et al., 2020).

#### 3.2.2 As carriers

Diversities in compositions and structures of natural plants offer a wealth of opportunities for botanicals as polymers in electrospinning. Natural polymers, such as lignin, guar gum, karaya gum, mucilage and pectin from hibiscus leaves have been used in electrospinning to improve physicochemical properties of wound dressings (Table 2). Gum arabic is a natural polysaccharide from the trunk exudate of *Acacia* trees. By using gum arabic and the corn protein zein, together with polycaprolactone polymer as a scaffold, studies have shown that the achieved hydrophilicity, elasticity, antibacterial potencies, and the cell penetration strength of nanofibers were mainly attributed to gum arabic, confirming that botanicals hold potentials to function as natural polymers for electrospinning in wound healings (Pedram Rad et al., 2018).

**TABLE 2:** Botanicals as carriers in electrospinning for wound healing.

Botanicals	Bioactive agent	Electrospinning design	Properties for wound healing	Advantages of botanicals	References
Lignin	-	Nanospider (needleless electrospinning)	1. Skin-friendly	1. Easy to access	Morganti et al. (2017)
			2. Acting on the modulation of matrix metalloproteinases, cytokines, and human beta-defensin 2	2. Biodegradability	
				3. Reducing the dependence on fossil fuel resources	
Guar gum	Paramagnetic iron oxide Fe_3_O_4_ nanoparticles	Blend/fibers obtained from alkaline stock solutions	1. Enhanced nanoparticle homogeneity	1. Hydrophilic	Lubambo et al. (2015)
			2. Increased nanoparticle stability	2. Non-toxic	
			3. Adequate levels of cytotoxicity and cell adhesion/proliferation	3. Biocompatible	
				4. Abundantly available in nature	
				5. Keeping long-term iron oxide nanoparticle stabilization through steric repulsion reducing both aggregation and sedimentation	
				6. The ability to chelate metal ions in alkaline pH	
Arabic gum/karaya gum/kondagogu gum	-	Nanospider/methane plasma treatment	1. Improved water contact angle	1. Non-toxic	Padil et al. (2016)
			2. High surface porosity and roughness	2. Hydrophilic	
			3. Superior hydrophobic properties	3. Acid stability	
				4. High viscosity	
				5. Potential antibacterial agent	
				6. Stabilizer and reducing agent in the synthesis of metal/metal oxide nanoparticles	
				7. Environmentally friendly	
Mucilage of *Hibiscus* leaves	-	Blend/crosslinked (glutaraldehyde)	1. Faster epithelization	1. Anti-bacteria: Gram-positive and Gram-negative bacteria	Sen et al. (2022)
			2. Hemocompatibility	2. Anti-oxidation	
			3. Biodegradability	3. Optimal skin moisturizing effect	
Pectin	-	Co-blended with polyethylene oxide, followed by selective washing off polyethylene oxide (containing only 1.5% polyethylene oxide)	A high Young’s modulus: 358.5 MPa	1. Anti-inflammation	Cui et al. (2016)
				2. Combination of ion-crosslinkable property	
				3. Biocompatible	
				4. Biodegradable	
				5. Hydrophilic	

#### 3.2.3 As coatings

In respect that some botanicals with excellent efficacy in promoting wound healing may not be suitable for electrospinning due to the exposure to harsh organic solvents, alternative modifications, such as coating, to directly immobilize botanicals into nanofibrous matrices have been proposed. Compared to methods that incorporate botanicals during electrospinning, the approach of coatings is more flexible and convenient in preparations (Albright et al., 2018). Moreover, physicochemical properties of the fiber surface could be easily remodeled by a cover of botanical envelopes, leading to promoted cell adhesion while reduced water evaporation (Jia et al., 2017; Richbourg et al., 2019). Aloe vera gel has wound-healing potentials due to its antioxidant, antibacterial, anti-inflammatory and anti-itch activities (Kang et al., 2014; Sánchez et al., 2020). In particular, the presence of carboxypeptidase and glucomannan in aloe stimulates fibroblast proliferation and promotes the synthesis and maturation of collagens (Nazeam et al., 2017; Gao et al., 2019; Zhang et al., 2021). The application of aloe gel as coatings for nanofibrous matrices has been attempted in l-polylactic acid (PLLA)-based nanofibers. By evaluating the healing process of mice with full-thickness skin defects, PLLA-coated with aloe had a faster onset of healing and an accelerated rate of wound-repairing compared to that without coating. Particularly, being highly hydrophobic, PLLA scaffold is prone to adhesions of *S. aureus*, the most important bacteria that cause human diseases, hence the covering by aloe vera gel simultaneously controlled potential bacterial infections (Jouybar et al., 2017).

### 3.3 Wound-healing effects of botanical electrospun nanofibers

#### 3.3.1 Hemostasis

Effective hemostasis is the prerequisite for wound healing. In addition to intrinsic mechanisms of hemostasis, ideal hemostatic materials that are efficient for blood clotting without causing secondary bleeding when being removed are clinically in need. Take the isoquinoline alkaloid berberine as an example. By embedding berberine in polycaprolactone nanofibrous membranes, a stronger capacity of blood clotting and a higher rate of platelet adhesion were observed. Moreover, the incorporation of berberine retained its antimicrobial potentials and improved the permeability and buffering properties of polycaprolactone matrix (Bao et al., 2013).

#### 3.3.2 Inflammation

During wound healing, adequate inflammatory responses ensure the transition of repair process to the phase of proliferation, whereas a persistent inflammation is detrimental. Shikonin, a naphthoquinone extracted from the root of *Lithospermum erythrorhizon*, has been loaded into polycaprolactone/polytrimethylene carbonate ultrafine fiber mats and exhibited antibacterial effects against *S. aureus* and *E. coli* compared to polymers alone (Han et al., 2009). Moreover, it was reported that an excessive ROS production by immune cells was harmful for wound healings, and applications of antioxidants are alternative ways to alleviate inflammation (Roy et al., 2006). For instance, the antioxidant fenugreek seed, which mainly contains trigonelline, naringenin, nicotinic acid, quercetin and saponins, has been introduced into nanofibers made of silk proteins to facilitate wound healings (Selvaraj and Fathima, 2017). Similarly, chrysin and grape seed extracts, which reduce oxidative stress, have been experimentally applied as wound dressings when loaded in polycaprolactone/polyethylene glycol or silk fibroin/polyethylene glycol, respectively (Lin et al., 2016; Deldar et al., 2018). Additionally, through regulations of pro-inflammatory cytokines, such as IL-1β and TNF-α, the artemisinin originally derived from the sweet wormwood (*Artemisia annua*), was incorporated into poly (lactic-co-glycolic acid)/silk fibroin and showed a shortened inflammatory cycle with enhanced skin regeneration in a rat model of dorsal full-thickness wounds (Peng et al., 2021). Likewise, by a direct effect on macrophages, ginsenoside Rg1 integrated into a novel asymmetric wettable electrospun membrane displayed a promoted macrophage polarization towards M2 phenotype, therefore facilitated a better infected wound healing compared to commercial Aquacel Ag dressings (Zhou et al., 2022).

#### 3.3.3 Proliferation

The proliferation phase of wound healing is characterized by collagen deposition, angiogenesis, keratinocyte-associated re-epithelialization, and fibroblast-related granulation tissue formation. In particular, the re-epithelialization and granulation tissue formation are the determining factors (Beavers et al., 2015; Martin and Nunan, 2015; Chattopadhyay et al., 2016; Foster et al., 2021). The loading of beet roots extracts *Beta vulgaris* into nylon 66 polyamide was shown effective in promoting the differentiation of keratinocyte-mesenchyme stem cell to epithelial linage during the healing process, which was associated with increased expression of cytokeratin 10, cytokeratin 14 and loricrin (Hosseinzadeh et al., 2017). When loading *Calendula officinalis* to polycaprolactone/zein/gum arabic, the polycaprolactone/zein/gum arabic/*Calendula officinalis* nanocomposite enhanced the proliferation and adhesion of fibroblasts for regenerating skin by mechanisms related to collagen production (Pedram Rad et al., 2019). Consistently, the *Calendula officinalis*-loaded chitosan/PEO nanofibers revealed excellent wound healing capabilities through enhanced collagen synthesis and deposition (Kharat et al., 2021).

#### 3.3.4 Remodeling

Hypertrophic scar formed by excessive cell proliferation during the healing process constitutes a serious issue that has great impacts on the life quality of patients (Finnerty et al., 2016). Current studies have shown that the formation of hypertrophic scars is closely related to excessive inflammation, overactivation of fibroblasts, and excessive proliferation of blood vessels (Wilgus, 2019; Wang et al., 2020b; Vorstandlechner et al., 2021; Chen et al., 2022). Based on the evidence that 20(R)ginsenoside Rg3 is effective in inhibiting the production of pro-inflammatory cytokines and down-regulating VEGF expressions, Rg3 was incorporated with polylactic acid as wound dressings (Seo et al., 2008; Tang et al., 2018; Ma et al., 2021; Nakhjavani et al., 2021). *In vivo* evidence showed that Rg3-containing membranes inhibited the early formation and late proliferation of hydrotropic scars, as demonstrated as reduced proliferation of fibroblasts, and minimized thicknesses of dermis and epidermis (Cheng et al., 2013).

## 4 Conclusion and future perspectives

Electrospinning has been involved in a broad range of applications, showing great potentials in regenerative medicine, gas filtration, food packaging and environmental purification (Chen et al., 2018; Chen et al., 2020; Das et al., 2020; Min et al., 2022). With continuous endeavors in developing botanicals into electrospun products, the proportion of electrospun wound dressings with medicinal plants in fundamental research is progressively increasing. Compared to synthetic polymers or pharmaceutical drugs/coatings, natural materials have better biocompatibility, bioactivity, and biodegradability, though drawbacks such as compromised flexibility and mechanical strength have been reported (DeFrates et al., 2018). Moreover, owing to their versatility in bioactivities, no or fewer side effects, and environmental friendliness, botanicals may create more possibilities for clinical applications, including but not limited to wound healings. In particular, the conventional bulk electrospinning machines have been upgraded to clinically available hand-held devices, which reduce difficulties in the access to electrospinning, therefore allows an immediate application of dressings according to a specific condition of injured skin (Yan et al., 2019).

At present, investigations in this field mainly focus on different methods in the preparation of botanical nanofibers, whereas their translation into clinical remains unaddressed. To put forward the clinical application of botanical-containing electrospun membranes, several aspects require additional efforts. Firstly, even though the bioactivities of a certain number of botanicals have been reported, their mechanisms of action kept largely uncertain. Therefore, a molecular level exploration of the individual compound in the process of wound healing needs to be emphasized. Secondly, since the wound healing process in clinics involves an orchestration of different cells and molecules, a continuous screening of herbals and their derivatives suitable for wound dressings is of particular importance in the context of complex clinical situations. Thirdly, in addition to effectiveness in wound healing, the improvement in practical issues, such as moisturization and tensile resistance remain to be solved. Fourthly, besides the necessity to formulate criteria for wound dressing, the establishment of standard or comparable experimental models appear to be primarily urgent. Finally, the commercial value of botanical electrospun nanofibers needs to be further reinforced, and the technology for a large-scale production should be developed along with the progress in the basic research.
